# Recording thoughts while memorizing music: a case study

**DOI:** 10.3389/fpsyg.2014.01561

**Published:** 2015-01-23

**Authors:** Tania Lisboa, Roger Chaffin, Alexander P. Demos

**Affiliations:** ^1^Centre for Performance Science, Royal College of MusicLondon, UK; ^2^Department of Psychology, University of ConnecticutStorrs, CT, USA

**Keywords:** memorization, performance, learning, teaching, thoughts

## Abstract

Musicians generally believe that memory differs from one person to the next. As a result, memorizing strategies that could be useful to almost everyone are not widely taught. We describe how an 18-years old piano student (Grade 7, ABRSM), learned to memorize by recording her thoughts, a technique inspired by studies of how experienced soloists memorize. The student, who had previously ignored suggestions that she play from memory, decided to learn to memorize, selecting Schumann’s “Der Dichter Spricht” for this purpose. Rather than explicitly teaching the student how to memorize, the teacher taught her to record her thoughts while playing by marking them on copies of the score, adapting an approach used previously in research with experienced performers. Over a 6½ week period, the student recorded her thoughts during practice (five times) and while performing from memory for the teacher (three times). The student also video-recorded 3 weeks of practice, three performances, and the reconstruction of the piece from memory after a 9½-weeks break. The thoughts that the student reported were prepared during practice, stable over time, and functioned as memory retrieval cues during reconstruction. This suggests that the student memorized in the same way as the more experienced musicians who have been studied previously and that teaching student musicians to record their thoughts may be an effective way to help them memorize. The speed and durability of her memorization surprised the student, inspiring her to perform in public and to use the same technique for new pieces.

## INTRODUCTION

Although playing from memory has a long history in Western classical music, there is little agreement among musicians about how memory for performance works or how to ensure its reliability (Aiello and Williamon, 2002). Many musicians appear to believe that memory differs so widely from one person to the next that it is best to leave each person to discover their own methods for memorizing (Ginsborg, 2002). As a result, memorization strategies that could be useful to almost everyone (e.g., Shockley, 1986) are not widely taught. Individual musicians know the strategies that work for them (Hallam, 1995, 1997) and some, no doubt, pass this knowledge on to their students. The musical community as a whole, however, does not advance. This is regrettable. Memory varies no more from one person to another than any other trait or capacity. Beneath a superficial diversity, the cognitive and neurological systems involved are common to all human beings (Rubin, 2006; Chaffin et al., 2009). Training has powerful effects on memory that have been studied for many years and are well-understood (Ericsson and Kintsch, 1995). Here we draw on studies of how experienced concert soloists memorize (Chaffin and Logan, 2006; Chaffin, 2011) in order to ask whether the methods used by experienced performers might be helpful to students learning to play from memory.

One practice technique commonly used by students is to start repeatedly from the beginning of the piece and play through to the end (Hallam, 1997; Renwick and McPherson, 2000; Lisboa, 2008). This produces rote memorization; each passage reminds the musician of what comes next in an associative chain. Associative chains develop rapidly and spontaneously and are remarkably accurate (Rubin, 1995, 2006). Unfortunately, they have the major drawback that you can only start in one place, at the beginning. For this reason, it is risky to rely exclusively on associative chaining. If memory fails and the chain breaks, the performer must start over (Chaffin et al., 2009). This kind of embarrassing memory failure is an unfortunate staple of student recitals. Students often make the mistake of assuming that, because they can get through the piece without the score in the studio, their associative chains can be relied upon in live performance. The associative chain is, however, just one step toward secure memorization, which requires different practice strategies and a lot more work.

Experienced performers rarely stop and go back. They know that memory failures are inevitable in live performance and they normally prepare a safety net that allows them to recover gracefully. They have a mental map of the piece that allows them to keep track of where they are as the performance unfolds. The map provides landmarks where they can restart the performance if necessary (Chaffin et al., 2002, Chap. 9). When something goes wrong, they jump to the next landmark and the performance continues. Most of the time, the audience is not even aware that anything went wrong. Creating this kind of safety net requires developing content addressable access to memory. Simply thinking of a passage brings it to mind. The thought, “G section,” brings to mind the sounds, movements, and feelings associated with playing it.

Content addressable access is provided by performance cues (PCs) embedded in a hierarchical organization based on the musical structure (Chaffin et al., 2002). PCs are what the performer thinks about during performance, e.g., “with feeling,” “sing,” “softer,” “repeated note.” They provide a mental map of the music that allows the performer to monitor the performance as it unfolds and to recover from mistakes and memory lapses. A PC is prepared by repeatedly thinking about a particular feature of the music during practice so that it comes to mind automatically during performance, directing the musician’s attention, and providing the possibility of consciously directing movements that would otherwise occur automatically.

Unfortunately, thinking about a skilled movement is a sure way to disrupt it, a phenomenon known in sports as “choking” (Beilock and Carr, 2001). When starting to play from memory, the disruption to the automatic motor sequences created by thinking about what you are doing is a substantial obstacle that must be overcome by practice, requiring time, patience, and persistence. This is what most people mean by “memorizing.” It is a slow and arduous process in which the serial chain of associations is integrated with content addressable access. Initially, content addressable access is much slower than serial chaining and it takes extended practice to bring it up to speed (Ericsson and Kintsch, 1995). Even when this goal is achieved, musicians still have to guard against the hands “running away” as the chain of motor sequences outstrips slower content addressable access to conceptual memory (Chaffin and Imreh, 2002). So long as the two forms of memory remain coordinated, however, content addressable access provides the safety net needed for reliable performance.

This understanding of performance memory is based on longitudinal case studies in which experienced performers recorded their practice as they prepared new works for public performance (see, Chaffin and Logan, 2006; Chaffin, 2011; Ginsborg and Chaffin, 2011 for reviews). The musicians provided detailed reports of each feature of the music that they had paid attention to during practice by marking them on copies of the score. Shortly after public performance, they marked additional copies of the score with the musical and technical features that they had thought about during the performance – their PCs. Comparison of the two sets of reports confirmed that during practice the musicians had paid attention to the places that they later attended to in performance (Ginsborg et al., 2012). Repeated reports from successive performances demonstrated the stability of PCs over time (Chaffin et al., 2013). Examination of the recorded practice showed that the musicians had begun building the mental organization needed to perform from memory in their earliest practice sessions, well before they were ready to try playing from memory (Chaffin and Imreh, 2002). Asking the musicians to play or write out the score long after the last public performance demonstrated that the musicians’ memories endured over periods of years (Chaffin and Imreh, 2002; Chaffin et al., 2010).

As one of the participants in these longitudinal case studies, the first author of the present study was impressed by the benefits of the method to her own work as a cellist (Chaffin et al., 2010; Lisboa et al., 2011). Talking to the camera as she practiced, and marking practice decisions and PCs on the score (part of the data collection method) made her more aware of her own musical intentions and the strategies she used to achieve them. Although participation in the research was time consuming, she found that her practice became more efficient and her confidence in her memory increased as a result, not only for the piece under study, but also for other pieces. Other musicians have reported similar benefits (Chaffin et al., 2002, pp. 266–269).

We suggest that the benefits that the first author noticed were a result of her increased metacognitive awareness of her goals and strategies for practice and memorization. Metacognition is a critical component of both thinking and learning (Veenman and van Hout-Wolters, 2006) and its contribution to effective study has received a great deal of attention from educators (Zimmerman and Schunk, 2011), including those in music education (see, McPherson and Zimmerman, 2002 for a review). The effectiveness of practice increases with experience because more experienced musicians are better able to assess their progress and adjust their practice accordingly (Jorgensen, 2004; Jorgensen and Hallam, 2009). The ability to self-monitor and self-regulate develops gradually. Initially, children often simply play through a piece repeatedly without noticing or correcting mistakes (Hallam, 1997; McPherson and Renwick, 2000; Lisboa, 2008). Advanced students and professionals, in contrast, monitor their playing continuously and adjust their practice strategies accordingly from moment to moment (Hallam, 1995; Nielsen, 2001; Chaffin et al., 2002; Chaffin et al., 2010, Chap. 6; de Graaff and Schubert, 2011).

Deliberate memorization appears to benefit from the additional reflection and self-monitoring involved in reporting PCs, at least for experienced performers. We wondered if student musicians could benefit in the same way. An opportunity to explore this question arose when a student of the first author’s (subsequently referred to as “the teacher”) decided that she would like to learn how to memorize a piece. Previously, the student had refused to work on memorization or to perform from memory. She had sometimes memorized incidentally, while learning a piece, but the memory was soon gone. PC-theory suggested that what the student lacked was a retrieval organization to provide her with content addressable access to the serial chain of associations that develops automatically during practice. The teacher’s experience suggested that making PC reports might help the student to develop a mental map to provide her with content addressable access, leading to more permanent and secure memorization.

Despite the well-documented positive effects of self-monitoring and self-regulation, it was not obvious that reporting PCs would have the desired effect. First, thinking about highly practiced motor skills is often disruptive (Beilock and Carr, 2001). The student might become discouraged if doing PC reports initially interfered with fluent, well established motor sequences. Second, we did not know if a student could benefit from thinking about PCs in the same way as the teacher, who was a professional soloist with decades of experience. While professional performers have no trouble identifying mental landmarks in a memorized piece, students are able to identify remarkably few (Aiello, 2001). Third, even highly experienced musicians find reporting PCs burdensome. Only in the latter stages of her self-study, did the teacher come to appreciate the benefit provided by the additional effort involved (Chaffin et al., 2010; Lisboa et al., 2011). It was possible that the student would give up before experiencing any benefits. In spite of these uncertainties, the student agreed to try the new method.

The teacher asked the student, who we will call “Maria,” to report her thoughts during practice and after performing from memory for the teacher during lessons. The teacher explained to Maria that she had found that reporting her own thoughts during the longitudinal case study improved her ability to practice and memorize; she expected that it would do so for Maria. The teacher offered no explanation as to why reporting thoughts would have these positive effects and avoided any mention of PCs. There were three reasons for this indirect approach. First, the teacher thought that showing the student what to do would be more effective than elaborate verbal explanation, which might be more confusing than illuminating. Second, the teacher tried to provide Maria with a discovery experience similar to her own during the case study. During that study, the teacher had avoided reading about previous research on PCs and had learned for herself that reporting her thoughts clarified her thinking and provided landmarks for her evolving mental map of the piece. Third, the teacher wanted to avoid making any suggestion about the kind of thoughts to use as PCs. If Maria was able to benefit from using PCs, it seemed possible that the kinds of thoughts she would find useful would be different from those that her teacher might use. Unlike the teacher, Maria had only a rudimentary knowledge of music theory, a passing familiarity with Western art music, and little experience of performance.

We will describe the thoughts that Maria reported and examine four types of evidence to see if they functioned as PCs (Chaffin, 2011). First, were Maria’s thoughts during performance prepared during prior practice sessions? Preparation during practice is a defining characteristic of PCs. Second, were her thoughts stable over time? Having the same thoughts at the same locations repeatedly over a period of weeks would indicate that they were a relatively stable part of her playing, another characteristic of PCs. Third, did her thoughts during performance occur at the same locations as stops and starts in earlier practice sessions? In the studies of professional musicians, this was the main evidence that they used PCs. Fourth, was her memory more enduring than the temporary memorization that she had achieved in the past? This was an important goal for Maria and is part of the everyday meaning of “memorization.” To test the durability of her memory for the selected piece in this study, the teacher asked Maria to reconstruct the piece from memory 9½-weeks after the end of the study, during which time she had not played the piece. We looked at whether starts and stops during the reconstruction occurred at the same locations as the thoughts that Maria had reported during her last performance, 9 weeks earlier. This would indicate whether her PCs were retained over time and were employed during retrieval from long-term memory.

## MATERIALS AND METHODS

### THE STUDENT AND TEACHER

Maria had taken piano lessons since the age of 4 and now at the age of 18, as she prepared for the transition from high school to higher education, she wanted to be able to play a piece from memory. Maria had been a private piano student of the teacher and first author for 6 years and was preparing pieces of Grade 7 standard of the Associated Board of the Royal Schools of Music (ABRSM), in England. These examinations do not require playing from memory and Maria had never deliberately set out to memorize a piece. Until this time, she had occasionally memorized pieces incidentally, as an unintended by-product of learning to play it; but after a few weeks the ability to play without the score would be gone. Now, she wanted to memorize a piece in order to audition for a place at a music academy or, if she went to university, to have something that she could play for friends and family in years to come.

Maria came from a middle class family that enjoyed and appreciated the arts. Maria and her younger brother took piano lessons for fun. Maria also took drama, ballet, and tap dance lessons. She limited her piano practice to 2–4 h a week. At the time of the study, Maria earned A-level grades in her chosen subjects of Geography (A), History (A), Maths (B), and Arts (A^∗^), the asterisk indicating outstanding exam performance. After a gap year, Maria went to university to study architecture. At time of publication, Maria was in her third year of study, still played the piano occasionally, and enjoyed going to concerts and listening to music.

The teacher was trained in classical cello and piano in Brazil, England and France and performs regularly as a soloist in Europe, the Far-East and the Americas. She has taught private students of all ages for more than three decades, including many, like Maria, who were not enrolled in music academies. As a Research Fellow at the Royal College of Music for the past decade, she has studied effective practice techniques by observing both her own playing and that of children.

#### Choice of Music

**FIGURE 1:**
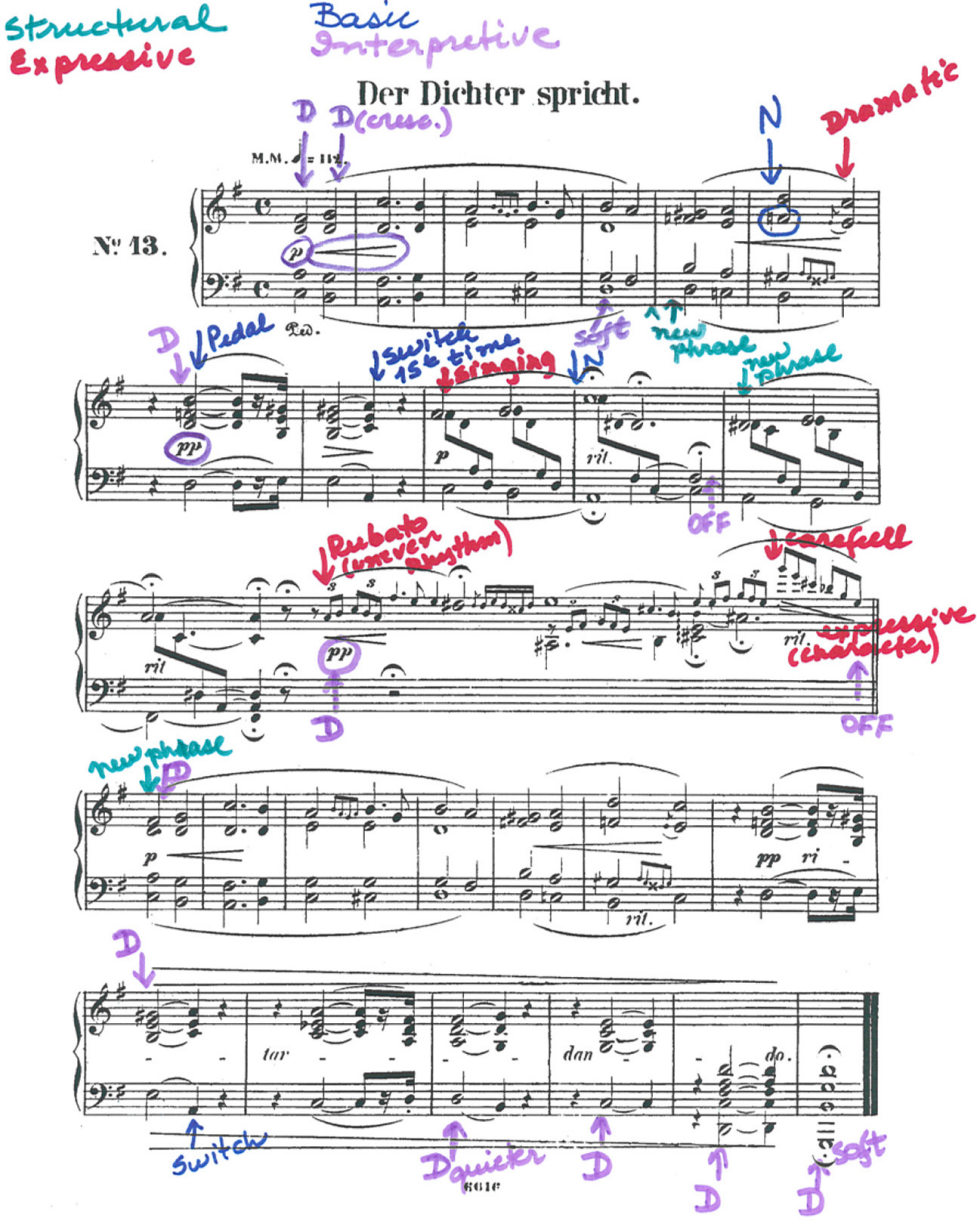
**“Der Dichter Spricht” (The Poet Speaks) from R. Schumann’s Kinderszenen Op.15 showing thoughts reported during practice preceding Lesson 5 (Report 5 in Table 1; red = expression, purple = interpretation, blue = basic, green = structural)**.

“Der Dichter Spricht” (The Poet Speaks) from R. Schumann’s Kinderszenen Op.15 (see **Figure 1**) is scored in 25 bars in common time, with a composed cadenza at the midpoint, bringing the total number of beats to 114. Teacher and student together chose this piece for Maria to learn and perform from memory because she found its lyrical and romantic style very attractive. Maria had briefly worked on “Der Dichter Spricht” a year earlier, but had put it aside as too difficult. In the meanwhile, her playing had progressed and the piece was now more appropriate to her skills. At the beginning of the study, Maria was able to play through the piece with the score but did so haltingly and with extremely limited musical expression.

#### Procedure

Following the choice of “Der Dichter Spricht,” Maria began to include the piece in her weekly practice with the new goal of memorizing it. During the first lesson of the study, the teacher showed Maria how to mark a copy of the score with arrows to indicate the features of the music that she was paying particular attention to during her weekly practice. At this point, Maria made a retrospective report of her thoughts and focus of attention during the previous week’s practice. Maria told the teacher which features of the music she had attended to and the teacher recorded them on a clean copy of the score, annotating each with an arrow to indicate which aspect of the music was involved, and using different colored inks to represent the classification of each feature as involving musical structure (e.g., “phrase”), expression (e.g., “dramatic,” “singing”), interpretation (e.g., “dynamics”), or basic technique [e.g., “hand position (HP),” “note”], following the same procedure the teacher had used in her own longitudinal case study. A sample report is shown in **Figure 1**. The teacher retained all reports at the end of each lesson, and Maria used a fresh copy of the score for each report. At the end of the study, the teacher provided an additional report, marking a copy of the score to show Maria’s phrasing by identifying the start of each phrase.

Over a period of 6½ weeks, Maria had seven lessons, practicing the piece at home in between lessons. During this time, she gave five reports of her thoughts during practice and three reports of her thoughts during three of the four memorized performances of the piece during lessons – a total of eight reports. **Table 1** provides a timeline organized in terms of lessons, which took place each week except during week 4, when there were two lessons. The table shows which lessons were preceded by practice (Lessons 1–6), which lessons included a performance for the teacher (Lessons 4–7), and for which of these activities Maria provided reports (Reports 1–8). The right hand three columns enumerate the three kinds of playing (practice, performances, reconstruction) as a single sequence of sessions in order to align the information in **Table 1** with the practice shown in **Figure 6**, which shows all of Maria’s playing that was recorded. Sessions that were recorded are listed in boldface in **Table 1** (Sessions 4–6 and 8–11).

**Table 1:** Timeline of lessons, practice, performances, and reports.

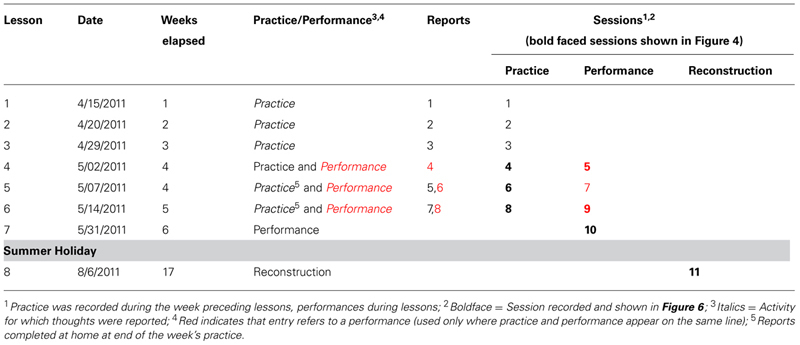

During Lessons 1–3, Maria reported her thoughts during the previous week’s practice to the teacher who marked them on copies of the score (Reports 1–3). During Lesson 4, Maria performed the piece from memory for her teacher for the first time. After the performance, student and teacher used the same procedure to make Maria’s first report of her thoughts during a performance (Report 4). The following week, Maria completed the report on her practice by herself (Report 5). She brought it to the next lesson (Lesson 5) and the student and teacher went through the report together, with the teacher annotating the report with different colors representing the classification of each feature as described above. During Lesson 5, Maria again performed for her teacher and, with the teacher’s help, completed another report of her thoughts (Report 6). During the next lesson (Lesson 7) Maria delivered her last report of thoughts during practice to the teacher (Report 7). She also performed for the teacher again, and made her final report of her thoughts during performance (Report 8).

Shortly after Lesson 7, summer holidays brought lessons to an end for several weeks. After the holiday, the teacher asked Maria to try to play “Der Dichter Spricht” from memory again and video-recorded her efforts. At this point, Maria had not played the piece for 9½ weeks and was unable to play without stopping. Starting and stopping, she worked her way through the piece from memory and then did so a second time, with greater fluency. These reconstructions from memory marked the end of the study.

Maria video-recorded 3 weeks of practice and three performances. Beginning during week 4, she delivered the recording of each week’s practice to the teacher at her lesson. Week 4 was also the week during which she gave her first performance for the teacher, which she did without the score during Lesson 4. Recording of practice during the week and performances during the lesson continued for two more weeks, through Lesson 6. At this point, Maria and her teacher concluded that the piece was memorized and Maria stopped recording her practice. Work on “Der Dichter Spricht” concluded with a final performance during the next lesson (Lesson 7), which was not recorded.

We transcribed the video-recorded practice, performances, and reconstruction by recording the location (in beats) of each start and stop. We also transcribed the reports by recording the location (in beats) of each feature reported, making separate tallies for features involving structure, expression, interpretation and basic technique. We used SYMP, an Excel-based software tool (Demos and Chaffin, 2009), to create graphical summaries of practice (Figures 6 and 7) and to relate the practice to the reports.

This study was granted ethical clearance at the Royal College of Music and was conducted according to ethical guidelines of the British Psychological Society. Informed consent was obtained from the participant, and no payment was given in exchange for participation.

#### Analysis

We evaluated the hypothesis that Maria’s reports were related to each other and to her playing against the null hypothesis that the different reports and her playing were unrelated to each other, calculating the probability that thoughts and starts while playing occurred in the same location by chance. In calculating these probabilities, we took the conservative step of excluding beats on which notes were held, judging it unlikely that thoughts would be reported or that playing would start at these locations (i.e., during rather than at the beginning of long notes). Rather than basing probabilities on the entire 114 beats of the piece, we based them on the 71 locations where the start of a note might trigger a thought or a new start in playing. The probability values (see **Table 2**) were generated from hypergeometric distributions (sometimes called a one-way Fisher’s test). Note that in graphing practice we have used 114 beats and we use beat numbers (1-114) to identify locations in the piece.

**Table 2:** Percentage of thoughts in each performance also reported in preceding practice sessions and of thoughts in practice retained in subsequent performance.

Performance	Preceding practice sessions	Thoughts in performance prepared in practice (%)	Thoughts in practice retained in performance (%)
1	**1–3**	88.9***	50.0***
2	**1–3 and 6**	87.5*	18.4*
3	**1–3,6 and 8**	100***	29.3***

*p < 0.05, ***p < 0.001.

We used generalized mixed effects models to evaluate the relationship of reported thoughts to starts during practice and reconstruction. Generalized mixed models are a type of regression model that can include discrete fixed factors (such as types of thoughts), continuous fixed factors (such as the number of starts and stops in practice), and crossed-random factors (such as locations of thoughts within the piece and the sequence of practice sessions). Mixed models allowed us to simultaneously control for differences between musical locations and differences between practice sessions providing a conservative method of controlling for changes in both (Singer and Willett, 2003). In our models, we treated starts as if they followed a Poisson distribution.

For practice, we drew predictors representing Maria’s thoughts from the reports for the performance that most immediately followed each practice session. (Thus, reports 4, 6, and 8 provided predictors for practice Sessions 4, 6, and 8 respectively). For the reconstruction, we drew predictors from report 8, the third performance report. We drew three predictors from each report to represent the location of PCs for basic, interpretive, and expressive features of the music. We also included starts of phrases (reported by the teacher) as a predictor. Thoughts and starts of phrases were coded by binary predictors with beats that were not the location of the start of a phrase or a PC assigned a value of zero. PCs and phrases were treated as fixed effects, as were the effects of practice sessions (three consecutive weeks of practice) and attempts (two attempts to play through the piece during reconstruction from memory). Locations where thoughts could occur were included as a random effect. In addition, practice sessions and attempts were also treated as random effects. The random effects were crossed as each session contained the same musical material. The estimated values that we report in the Tables 4 and 5 are the expected (natural) log count and probability values based on *z*-values created from the estimated value divided by the SE value. Degrees of freedom are not provided in mixed models (see Bates et al., 2014).

## RESULTS

We begin by describing examples of the thoughts that Maria reported, showing how their content changed over time. Then we look at evidence that the thoughts that Maria reported had the characteristics expected of PCs. In Section “Preparation of Thoughts in Practice,” we ask whether the thoughts that Maria reported during performances were also reported for the practice sessions preceding each performance. In Section “Stability of Thoughts Over Time,” we ask whether Maria’s thoughts were stable over time: Did she report thoughts in the same places from one report to the next? In Section “Comparing Thoughts with Starting Places in Practice,” we examine Maria’s practice to see if she stopped and started at locations where she reported thoughts during the preceding week’s practice. Finally, in Section “Comparing Thoughts with Starts and Stops During Reconstruction,” we do the same for the reconstruction from memory, asking whether the places where Maria stopped and started were the same places where she had reported thoughts during her last performance.

### DESCRIPTION OF REPORTED THOUGHTS

As with any highly skilled activity, most of Maria’s playing was automatic. She reported thoughts about the music at only a minority of the possible locations in the piece that afforded opportunities for thinking. Of the 71 possible locations, the number where she reported thoughts ranged from a maximum of 26 (29.9%) in Report 1 to a minimum of 10 (11.5%) in Report 6. The number of thoughts did not change reliably over time and was not significantly different for practice and performance.

**FIGURE 2:**
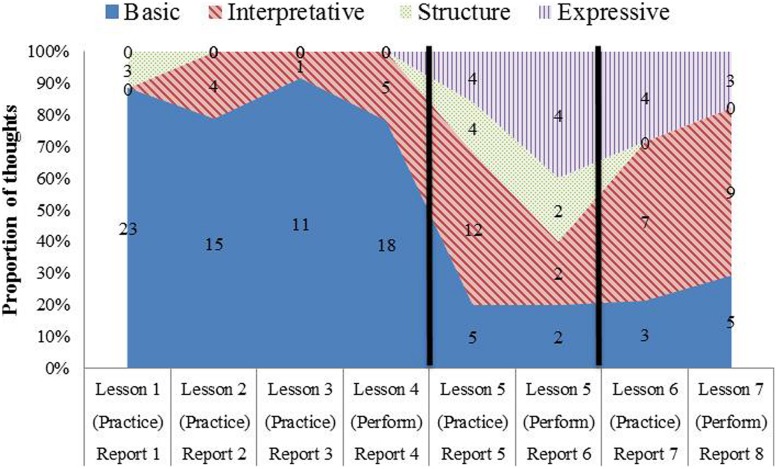
**Proportion (%) and number (superimposed values) of thoughts about four different aspects of the music contained in eight reports about practice and performances over a 5 week period which is divided into three phases (memorization, developing interpretation, and polishing) by the black vertical lines**.

The nature of Maria’s thoughts changed over time. **Figure 2** shows the proportion (and number) of thoughts about basic technique, interpretation, expression, and phrasing in each report. The proportions of the four types of thoughts in each performance were similar to the practice that preceded it. In contrast, after each of the first two performances, there was a substantial change in the proportions of the four types of thought. On the basis of these shifts and the teacher’s observation of Maria’s progress, we can divide Maria’s learning into three phases: Memorization (Lessons 1–4), developing interpretation (Lesson 5), and polishing (Lesson 6). The three phases are separated in **Figure 2** by vertical black lines.

The memorization phase came to an end in Lesson 4, when Maria surprised herself and her teacher by playing the entire piece from memory for the first time. Maria commented “I did not know I could play from memory...but I still need more work.” Her teacher noted that she not only played from memory, but also played more musically than usual, slowing at ends of phrases and using some dynamic contrasts. During this performance and in the practice that preceded it (Reports 1–4), Maria thought mostly about basic technique, focusing on hand position, individual notes and note sequences, and switches (places where repetition of the same musical material invited confusion). After this first performance, Maria’s attention shifted from memorization to interpretation (Reports 5 and 6). Basic thoughts decreased sharply, and thoughts about expression (e.g., feeling, singing) and phrasing reappeared for the first time since Report 1. A week later, after the second performance in Lesson 5 (Reports 7 and 8), there was another transition. Maria stopped thinking about phrasing, which apparently became automatic, allowing her to focus on dynamics and expression. After performing for her teacher in Lesson 6, Maria pronounced herself satisfied with her progress and suggested that it was time for her to set the piece aside and move on to other repertoire.

**FIGURE 3:**
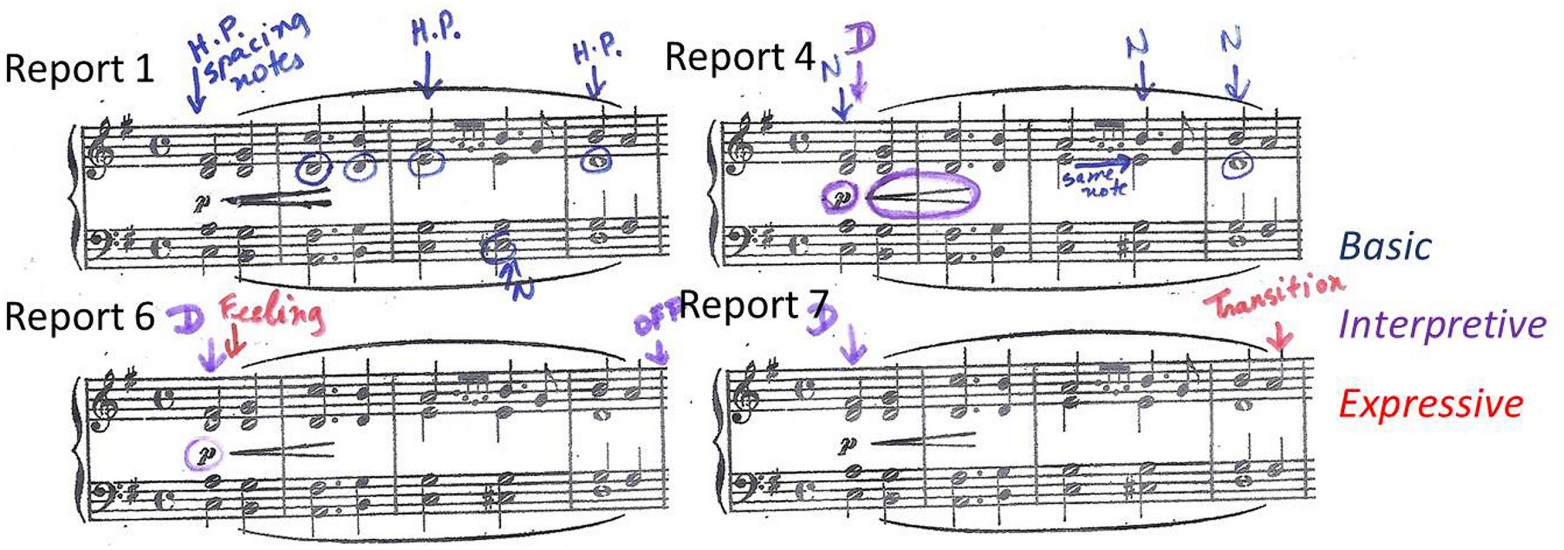
**Thoughts about bars 1–4 in Reports 1, 4, 6, and 7 showing progression from basic (blue) to interpretive (purple) and expressive (red) thoughts**.

An example of how Maria’s thoughts changed from basic to more expressive thoughts can be found in bars 1–4. **Figure 3** shows the thoughts about these bars in Reports 1, 4, 6, and 7. In Lesson 1, Maria reported that in order to learn and memorize the chords she was ‘focusing on the spacing of the hand.’ **Figure 3** shows how the teacher marked this in the score in Report 1 as ‘hand position’ (HP), with arrows pointing to the chords in question and with the problematic notes circled. By the time of her first performance, in Lesson 4, Maria’s attention at these locations had shifted from chords to specific notes. These are shown in **Figure 3** (Report 4), where the teacher marked ‘notes’ (N), circling those that Maria reported thinking about during the performance. In two cases, these are at locations where Maria had previously reported thoughts about hand position, suggesting that she was thinking about the same problem, but was now able to focus on the source of the problem more specifically. By this time, Maria was also becoming more aware of dynamics. Report 4 also shows that she was thinking about starting softly and then growing louder. The teacher recorded this by marking ‘dynamics’ (D), in purple to indicate ‘interpretation,’ and circling the relevant dynamic markings in the score.

After the first performance, thoughts about basic issues such as notes receded. Maria continued to think about interpretation and began to also think about expression. The change is evident in **Figure 3** (Report 6), made in Lesson 5 after the second performance. Maria’s earlier thought about the dynamics of the opening bar was repeated. In addition, Maria reported that she was thinking about the feeling she wanted to convey from the start of the piece. This is represented by the notation *‘feeling’* at the same location, in red to indicate expression. In her next report, Report 7, made after her next performance in Lesson 6, Maria continued to think about the dynamics of the opening. Here, however, her thoughts about musical expression had changed. Instead of thinking about the feeling conveyed by the opening crescendo, Maria reported that she was thinking about the need to ‘breathe’ in the transition from the first to the second phrase. The teacher recorded this as ‘transition’ at end of bar 4, using red for ‘expression’ to indicate Maria’s concern to create a change in the musical feeling.

**FIGURE 4:**
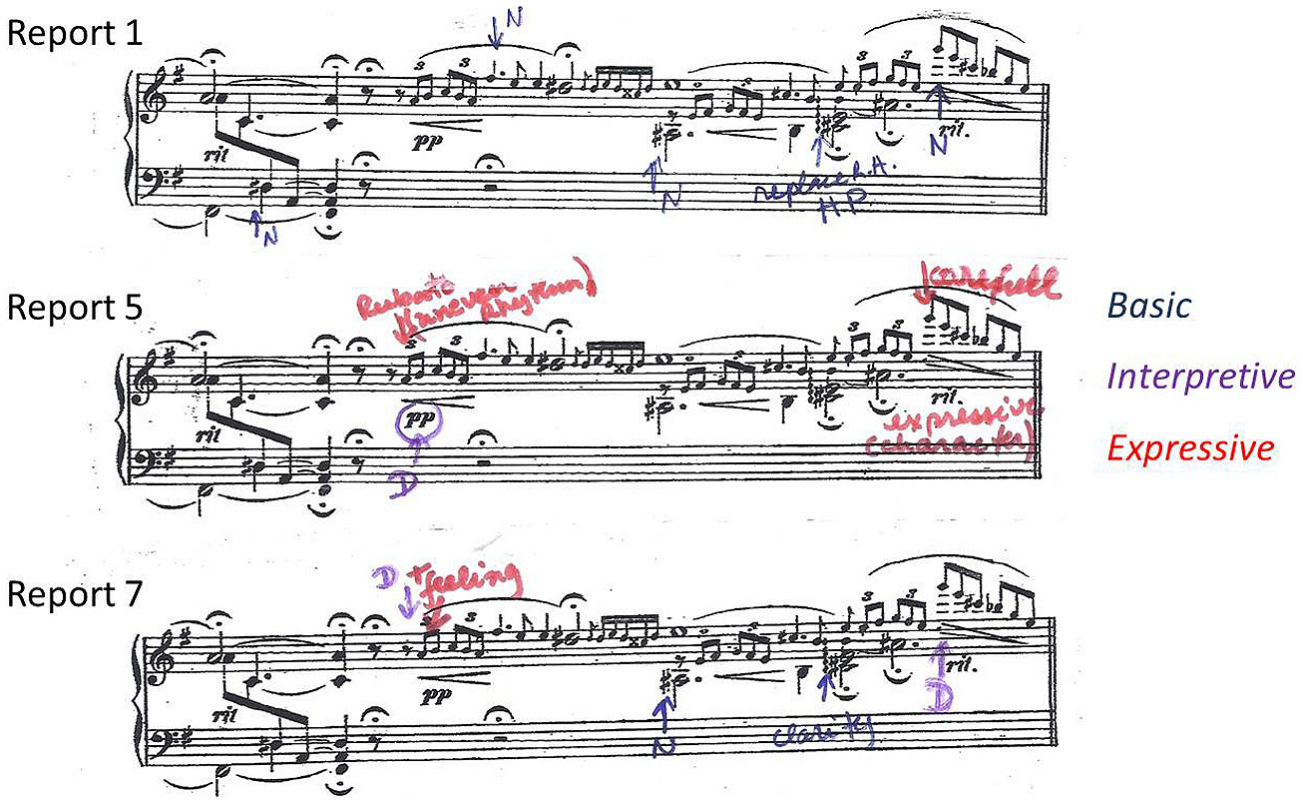
**Thoughts about bar 12 in Reports 1, 5, and 7 showing progression from basic (blue) to interpretive (purple) and expressive (red) thoughts**.

A more elaborate example of the same pattern of development of thoughts from basic to interpretive and expressive is provided by bar 12. **Figure 4** shows how Maria’s thoughts about the composed cadenza in bar 12 developed from basic issues concerning notes and hand position in Report 1 (Lesson 1) to dynamics and expression in Reports 5 (Lesson 5) and 7 (Lesson 6).

**FIGURE 5:**
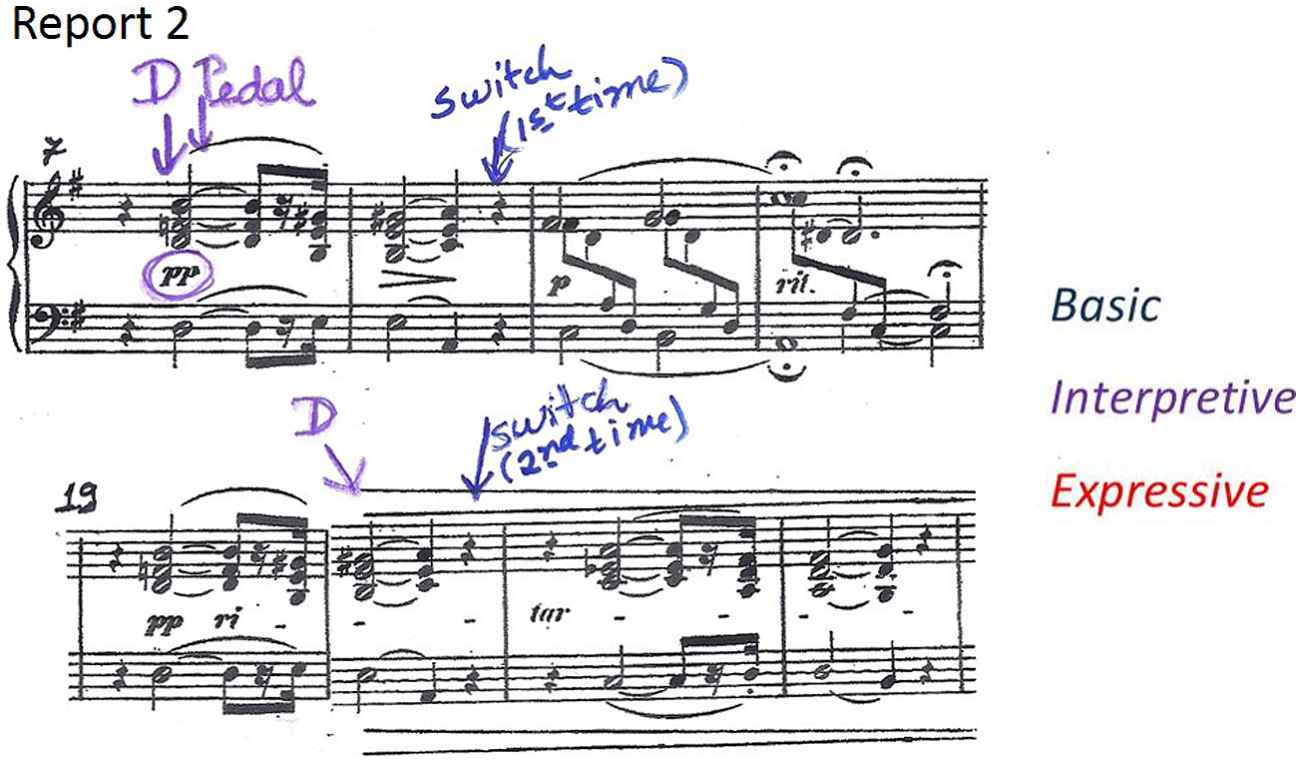
**Examples of a *switch,* marked in Report 2 (Lesson 2) at bars 8 and 20**.

Maria also reported thoughts about the danger of confusion due to the repetition of similar musical material in different locations in the piece (bars 7–8 and 19–20; see **Figure 1**). In Lesson 2, Maria reported that she was thinking *‘first time’* in bar 8 and ‘*second time’* in bar 20, in order to prepare for what came next, in bars 9 and 21 respectively. The teacher explained to Maria that experienced soloists also found it important to attend to such places and that memory researchers referred to them as ‘*switches.’* **Figure 5** shows how the teacher recorded thoughts about switches in Report 2 (Lesson 2). Unlike the experts, who reported switches in the bars where they occurred, Maria reported thinking about the switch a bar before it occurred, at the point where she prepared herself for the upcoming switch by anticipating it.

### PREPARATION OF THOUGHTS IN PRACTICE

The great majority of Maria’s thoughts during performance were prepared during prior practice sessions, and thus appear to have been PCs. **Table 2** shows the percentage of thoughts during performance that occurred at locations where she had previously reported a thought in practice and vice versa (columns 1 and 2 respectively). Across the performances, the great majority of thoughts (Mean = 92.1%) occurred at locations where thoughts had occurred previously during practice. It is very unlikely that this degree of overlap in locations would have occurred by chance. Thus, the thoughts at these locations were PCs, i.e., thoughts prepared during practice.

In contrast, many of Maria’s thoughts during practice did not recur during subsequent performances. The second column of **Table 2** shows that, across practice sessions, only a third of thoughts during practice (Mean = 32.6%) occurred at locations where thoughts later occurred in a subsequent performance. Thus, most of the musical features that Maria thought about during practice, she did not think about during performance. Appropriately, her performances were more automatic than her practice.

These values for the overlap of thoughts during performance and thoughts during practice and vice versa (92 and 33% respectively) are roughly comparable with those for the professional singer studied by Ginsborg and Chaffin (2012) and Ginsborg et al. (2012). In two separate studies, the singer prepared 51 and 61% of her thoughts during performance in prior practice and retained 46 and 47% of her thoughts during practice in subsequent performance. Substantial differences from the present study preclude direct comparison. (To count as the same, thoughts in Ginsborg’s (2002) studies had to be reported at the same location and also to be of the same type). Nevertheless, the overlap of Maria’s thoughts during practice and performance was roughly comparable to the singer.

### STABILITY OF THOUGHTS OVER TIME

A second type of evidence that Maria’s thoughts during performance were PCs is provided by the stability of their content over time. In order to summarize the stability of her thoughts over time, we classified them into four mutually exclusive categories: no thought, basic (technique) thought, interpretive/expressive thought, or both (the later indicating that both basic and interpretive/expressive thoughts were reported at the same location in the same report). We then tabulated the frequency with which the four types of thoughts succeeded one another in consecutive reports. **Table 3** shows the frequencies, across all eight reports.

**Table 3:** The percentage of thoughts in each report that reappeared in the following report, separately for four types of thought.

	Type of thought in later report			
Type of thought in earlier report	No thoughts	Basic	Interpretive/Expressive (I/E)	Both (Basic and I/E)
No Thoughts –>	91.1%	3.6%	4.1%	1.2%
Basic –>	43.3%	45.0%	8.3%	3.3%
I/E –>	50.0%	3.6%	35.7%	10.7%
Both –>	44.4%	11.1%	33.3%	11.1%

As already noted, Maria reported thoughts at only a minority of the locations in the piece where thoughts were possible, indicating that her playing was largely automatic. This is reflected in the large values in the No Thoughts column in **Table 3**. Not only did Maria report no thoughts at most locations, but also she was largely consistent in doing so, reporting no thoughts at the same locations 91% of the time. When thoughts did occur, they often disappeared, replaced by No Thoughts in the next report 43% of the time or more.

When thoughts did recur at the same location in successive reports they were, however, usually of the same type. Thoughts about basic issues were followed by thoughts about basic issues 45% of the time and by thoughts about interpretive issues only 8% of time. Interpretive/expressive thoughts were followed by interpretive/expressive thoughts 35% of the time, and by basic thoughts only 3% of the time. Thoughts about both (basic and interpretive/expressive) issues were followed by thoughts about one or other, or both, 55% of the time (11 + 33 + 11%). Thus, while there was considerable variability in whether thoughts occurred or not, when thoughts did occur, their content tended to be consistent.

The value of 35–55% stability is conservative because it reflects only stability across adjacent reports. It does not include thoughts that occurred intermittently, appearing in one report and not the next, and then reappearing again in a later report. For example, at bar 7, beat 2, Maria reported thoughts about dynamics and hand position (Report 2), dynamics and pedal (Report 4), pedal (Report 6), and dynamics (Reports 7 and 8), and No Thoughts (Reports 1, 3, and 5). This yielded a stability value of 0% based on the following sequence of classifications: No Thought, Both, No Thought, Both, No Thought, Basic, Both, Interpretive. The stability of her thoughts about dynamics and pedal was not picked up by our measure because they did not occur in adjacent reports. Our measure thus underestimates the stability of thoughts across all of the reports. Despite this, there was substantial stability from one report to the next.

### COMPARING THOUGHTS WITH STARTING PLACES IN PRACTICE

**FIGURE 6:**
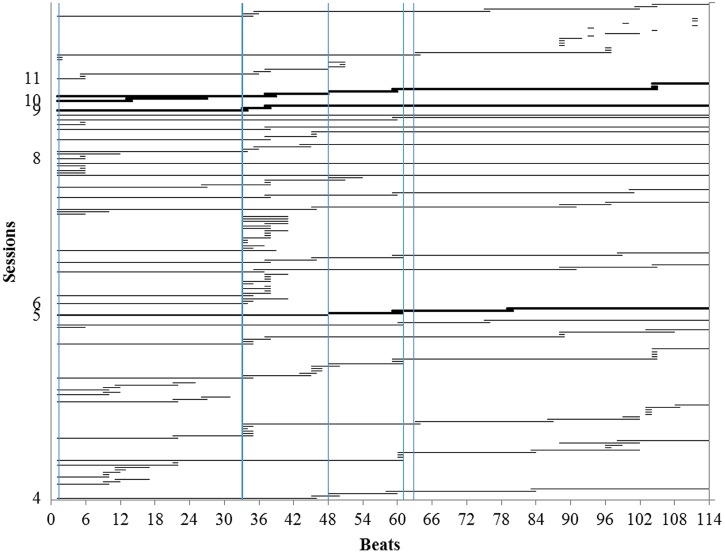
**Playing during practice and performances (thin and thick horizontal lines respectively).** The practice record reads from bottom to top with horizontal lines representing successive segments of uninterrupted playing. Instead of numbering the practice segments, the vertical axis shows the sessions in which they occurred. Vertical lines show locations of thoughts about expression during performances. (Only practice sessions that were recorded are shown here. These are listed in boldface in **Table 1**).

A third type of evidence that Maria’s thoughts during performance were PCs comes from a comparison of her reports and playing. **Figure 6** shows the three practice sessions and three performances that she recorded. The graph reads from bottom to top, with each horizontal line representing the uninterrupted playing of the beats shown on the horizontal axis below. Each time playing stopped and started again, the record begins again on the next line up. Practice is represented by thinner lines, performances by thicker lines.

Like other students, Maria’s practice consisted largely of playing through the piece from beginning to end, stopping only occasionally to single out short passages for more intensive work (Renwick and McPherson, 2000; Williamon and Valentine, 2002; Lisboa, 2008). Mostly, when she stopped, she started again at the same location, backing up a few beats to where the note began when necessary. This was true of both practice and performance, which were very similar. Playing started again at the same location where it stopped for 59.5% of stops during practice and 84.6% of stops during reconstruction. Instruction in effective practice strategies had long been a regular part of Maria’s lessons. When asked about her practice of this piece, she told her teacher that she did not simply play through from beginning to end but worked on it section-by-section. This was certainly not true of the practice in **Figure 6**. With the exception of one passage (around beat 36), Maria simply played through the piece, backing up briefly when she encountered problems. We cannot say whether Maria had a mistaken view of how she practiced or whether she practiced differently from usual in these sessions, perhaps trying for a fluent performance for the camera or because she was playing from memory. Whatever the explanation, the ineffectiveness of her practice strategy was evident in the performances. She never did give a fluent, uninterrupted performance. Instead, her three performances look very much like her practice.

The vertical lines in **Figure 6** represent the location of thoughts about expression during the three performances for the teacher. The intersection of vertical lines (representing thoughts) with the beginnings and ends of horizontal lines (representing playing) are places where thoughts coincided with starting or stopping. Every thought coincides with at least one start or stop, some coincide with many, e.g., beat 33. Starting at a particular location requires thought and creates an associative link between the thought and the playing that follows, creating a PC. The preponderance of intersections in **Figure 6** suggests that most, if not all, of the thoughts that Maria reported during performances were PCs. These were locations that she thought about during practice. Her thoughts at these same locations during performance were not, therefore, accidental or arbitrary. They were prepared during practice.

To determine whether Maria’s thoughts during performance intersected with starts during practice more frequently than expected by chance, we compared her thoughts during each performance with the starts during the practice session or sessions that preceded it. Specifically, we compared thoughts during the first performance (session 5) with starts during practice in Sessions 1–3, the second performance (Session 7) with practice in Session 6, and the third performance (Session 9) with practice in Session 8. We do not report the analyses of stops because they overlapped substantially with starts and were less strongly related to thoughts. We analyzed the reconstruction separately, and did not analyze starts and stops in the performances because they provided too little data.

**Table 4** summarizes the mixed model for practice. The significant effects for expressive thoughts and thoughts about basic technique indicate that playing started more frequently than expected by chance at locations where Maria reported these types of thoughts. There was also a significant effect for thoughts about interpretation, but the effect was negative, indicating that Maria started at these locations less frequently than expected by chance, i.e., she avoided starting at these locations. Like the positive effect for expressive thoughts, the negative effect indicates that Maria was thinking about these locations. Instead of starting, however, she played through them without stopping, providing “practice in context,” a strategy that has been observed in the practice of professional musicians (Chaffin et al., 2002, p. 187; Chaffin et al., 2010). The analysis thus confirms that Maria’s thoughts during performance about expression, interpretation and basic technique had been prepared during practice.

**Table 4:** Summary of mixed model of relation of thoughts during subsequent performance to starts during prior practice.

Fixed effects	Estimate	SE
Intercept	-2.66***	(0.53)
Expressive thoughts	2.31***	(0.56)
Interpretative thoughts	-1.21*	(0.53)
Basic thoughts	1.34**	(0.41)
Session	-0.72***	(0.16)
Phrase Starts	0.61	(1.13)

**Random effects**	**SD**	

Session	0.001	
Locations	2.17	
**Goodness of fit measures**
Akaike information criterion (AIC)	389.1	
Bayesian information criterion (BIC)	419.6	
Deviance [–2 (log likihood)]	373.1

*p < 0.05, **p < 0.01, ***p < 0.001.

Another way in which Maria’s practice was similar to that of professional musicians is that her practice sessions became shorter over time. This is reflected in the negative effect for sessions which indicates that there were more starts in earlier sessions. The same effect is seen in the practice of professional musicians (Chaffin et al., 2002, p. 127). One way in which Maria’s practice differed from that of professional musicians is that she did not use beginnings of phrases as starting places. Professional musicians, in contrast, organize their practice in terms of the musical structure which has large and consistent effects on starts during practice (Chaffin et al., 2002, pp. 190, 205–216; Chaffin et al., 2010).

Despite Maria’s practice of expressive, interpretive, and basic PCs, her performances were not fluent. We attribute this to her practice strategy of simply playing through the piece. As a result, her PCs did not receive the extended repetition needed to operate reliably. When content-addressable retrieval from long-term memory failed to keep up with the pace of performance, she was obliged to stop while she thought about what to do next. In most cases, she just needed a little more time to remember and then was able to continue from the same location. Bringing content addressable retrieval from long term memory up to the speed required for performance needed more practice (Ericsson and Kintsch, 1995; Chaffin and Imreh, 2002; Chaffin, 2011).

### COMPARING THOUGHTS WITH STARTS AND STOPS DURING RECONSTRUCTION

**FIGURE 7:**
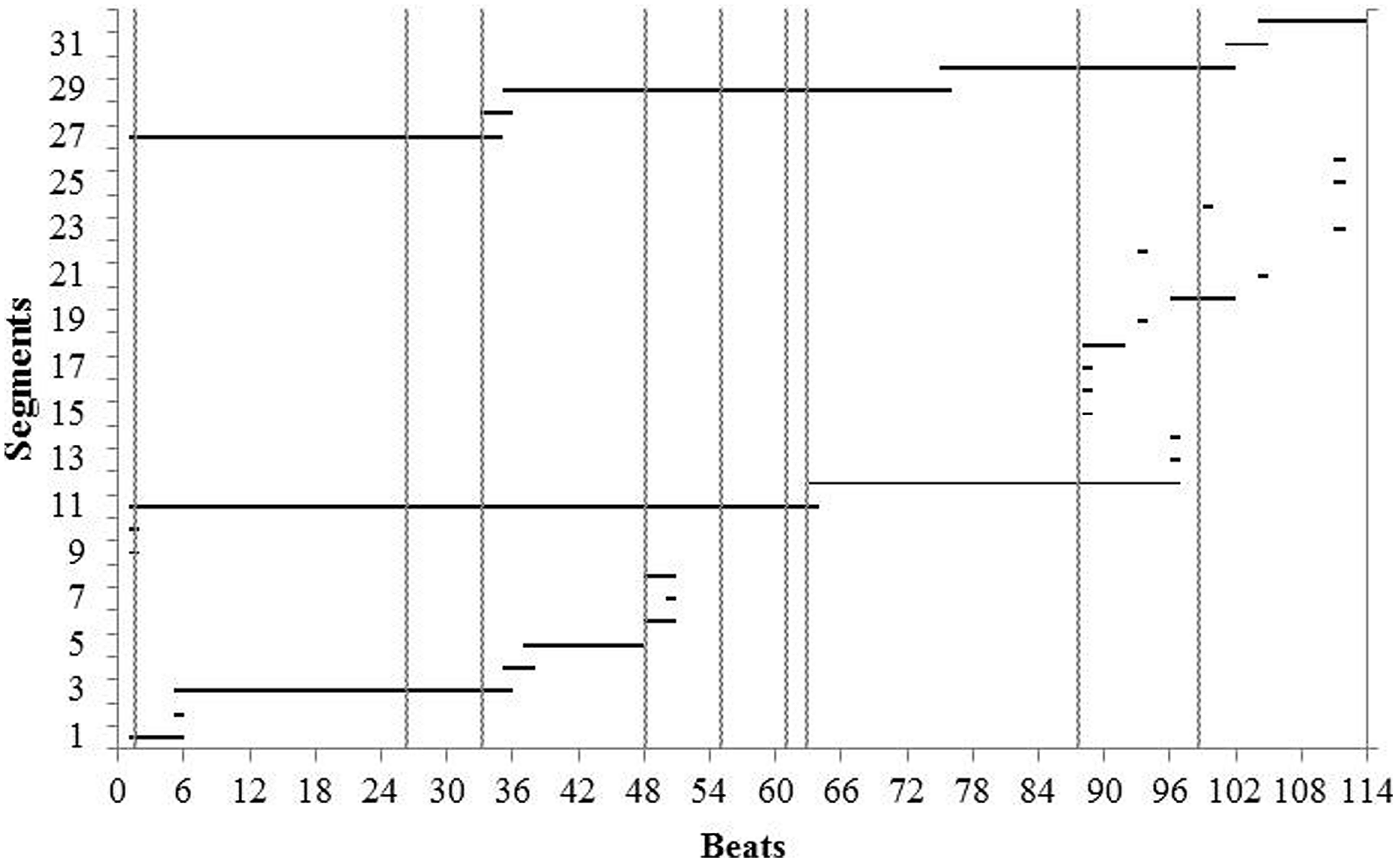
**Reconstruction of the piece from memory during Lesson 8, after not playing it for 9 weeks, showing the location of thoughts about interpretation (vertical lines) reported for the third performance.** The graph reads from bottom to top with horizontal lines representing successive segments of uninterrupted playing.

Finally, we looked at the reconstruction of the piece from memory when Maria resumed piano lessons after the summer break. We related the locations where she started to play to the locations where she had last reported thoughts during a performance (Report 8), 9½ weeks earlier. Maria’s attempts to reconstruct the piece from memory in Lesson 8 are shown in **Figure 7**, which also shows the location of thoughts about interpretation in the third performance (vertical lines). Three of these thoughts provided starting places during the reconstruction and thus appear to have functioned as PCs, providing content addressable access to her memory at the beginnings of three major sections of the piece: cadenza, second half, and final *ritardando* (beats 48, 63, and 88 respectively).

Beginning at the bottom of **Figure 7**, we see that Maria’s first attempt to play through the piece stalled at beat 49. She was able to re-start from beat 48, where she had earlier reported thoughts at the beginning of the cadenza about dynamics as well as about playing the “new phrase” with “rubato, uneven rhythm.” From this starting point, she made three attempts to go on before giving up on this first attempt and going back to the beginning. This marked the end of the first of the two attempts into which we divided the reconstruction for analysis.

At the beginning of the second attempt, Maria got through the cadenza successfully, only to stop at the beginning of the second half. Again, she found a starting point at a place where she had previously reported thinking variously about “dynamics,” as well as about the “new phrase,” and “feeling” (at beat 63). From here, she played without interruption until the final cadence, where she stopped again. She tried to continue, twice, and then retreated to the beginning of the final *ritardando* (beat 88), where thoughts about dynamics, reported for the third performance, provided another starting point. By this time, Maria had become flustered and was able to retrieve only isolated fragments for the remainder of the piece. She then started again at the beginning and played the piece one more time. (We treated this as a continuation of the second attempt because the suggestion to start over came from the teacher). This time she was able to get through the piece to the end, with no more stops and starts than in her earlier performances from memory. She had successfully reconstructed the piece from memory.

The analysis of starts during reconstruction, summarized in **Table 5**, confirms that starts occurred at locations where Maria had thought about interpretation during the third performance more frequently than expected by chance. The analysis also shows that she made fewer starts during the first than in the second attempt, reflecting the fact that the first attempt stopped at beat 49. There was also a non-significant trend to start at the same places during reconstruction that she had previously started during practice.

**Table 5:** Summary of mixed model of relation between thoughts during the third performance and thoughts during reconstruction of the piece from memory.

Fixed effects	Estimate	SE
Intercept	-3.33***	(0.87)
Expressive thoughts	-1.41	(1.90)
Interpretative thoughts	1.84*	(0.86)
Basic thoughts	1.09	(1.14)
Attempt	-1.49**	(0.50)
Phrase starts	0.33	(1.41)
Practice (Starts)	0.17^†^	(0.09)
Practice (Stops)	-0.02	(0.10)

**Random effects**	**SD**	

Attempt	0.0004	
Locations	1.54	
**Goodness of fit measures**
Akaike information criterion (AIC)	165.7	
Bayesian information criterion (BIC)	199.9	
Deviance [–2 (log likihood)]	145.7	

^†^*p* < 0.10, *p < 0.05, **p < 0.01, ***p < 0.001.

The places where Maria started during the reconstruction suggest that her thoughts during the third performance, 9½ weeks earlier, were PCs. When serial cuing failed her, Maria was able to back up to places where she had thought about interpretation in her last performance. For example, by thinking again “rubato, uneven rhythm,” she provided herself with a retrieval cue that allowed her to re-start her playing and go on.

## DISCUSSION

Maria’s thoughts while performing from memory for her teacher were PCs. First, her thoughts were prepared during practice. They were not about random features of the music that happened to catch her attention. They occurred at the same locations where she had thought about the music during the previous week’s practice, e.g., “feeling,” “dynamics,” “repeated notes.”

Second, her thoughts during performance occurred at locations where she had earlier started playing during practice. Starting created an associative link between the thought of the music and the actions involved in playing. Both characteristics suggest that Maria’s thoughts during performance were prepared during practice, the defining characteristic of PCs (Chaffin et al., 2002, 2010; Ginsborg and Chaffin, 2012; Ginsborg et al., 2012).

Third, Maria’s thoughts were relatively stable over time. Thoughts about the same locations reappeared on different occasions over a period of 7 weeks. There was variability from one report to the next: the same thought often appeared, disappeared, and then reappeared; and the nature of the thoughts at a particular location sometimes changed from one time to the next. Even so, Maria’s thoughts were no more variable than those of the two professional musicians whose thoughts during performance have been examined in this way (Ginsborg and Chaffin, 2012; Ginsborg et al., 2012; Lisboa et al., 2013).

Fourth, Maria’s thoughts about interpretation during her final performance served as retrieval cues when she came to reconstruct the piece from memory 9½ weeks later. This conclusion supports a central claim of PC-theory that PCs function as retrieval cues. Thoughts during performance not only direct attention to aspects of performance that need to be monitored, they also elicit memories for the upcoming passage from long-term storage. Direct evidence for this claim has been relatively scant. Two studies have shown that written recall of the score is better at expressive PCs and beginnings of sections many months after performance (Chaffin and Imreh, 2002; Chaffin et al., 2010). A third study has shown effects of PCs on reconstruction from memory months after performance (Ginsborg et al., 2012). The present results provide additional evidence that PCs aid memory retrieval.

PCs make it possible to recall specific passages in a piece of music by providing a mental address. By thinking of a location in the piece, the musician is able to recall the details of what happens there. This is what is meant by “content addressable memory” (Chaffin et al., 2009). Content addresses require the material to be organized in some way (Ericsson and Kintsch, 1995). For music, the organization is provided by the musical structure which experienced musicians use both to organize their practice and as a retrieval organization for their memory (Chaffin and Imreh, 2002; Chaffin et al., 2010; Ginsborg et al., 2012). Musical structure appears to have been less important for Maria than for the professional musicians in previous studies. Unlike the professionals, Maria did not start at beginnings of phrases during practice or reconstruction and she reported relatively few thoughts about structure.

We did not document Maria’s earlier memorization and cannot conclude that the positive outcome in our study was due to Maria reporting her thoughts. What we have shown is that Maria spontaneously began to use PCs as part of learning to memorize. She discovered this for herself. Her teacher did not tell her what to pay attention to, did not discuss the idea of PCs, and did not explain how reporting thoughts was expected to help with memorization. All the teacher did was to teach Maria how to report her thoughts, and motivate her to do so by telling her that she (the teacher) had found it helpful. Maria’s discovery of PCs echoes the experience of her teacher. The conclusion of both student and teacher that reflecting on their own thoughts while performing was beneficial is consistent with the large literature showing positive effects of metacognition on learning (McPherson and Zimmerman, 2002; Jorgensen and Hallam, 2009; Zimmerman and Schunk, 2011).

### THE PARTICIPANT’S REFLECTIONS

Four years after the study, we asked Maria about her past and current involvement with music. Her answers suggest that positive effects of participation in the study continued for a long time afterward.

‘At the time of the study, ballet was a big part of my life. Piano wasn’t as big as ballet, but probably comparable to tap dance. Now, University takes most of my time and I don’t have much time to play, nor do I have a piano in my flat. When I go to my Dad’s house, however, I try to play the things I remembered better and I enjoy it.’

When asked about which pieces she plays now, she replied:

‘*The Schumann is one of the pieces I like playing, because I know it better. I cannot play it from beginning to end from memory, without looking at the music first, but if I play it one time looking at the music, then I can play from memory even now! It was such a long time and I can still play it. This does not happen with other repertoire.’*

We asked how she currently practices:

‘Well, I don’t have time to practice anymore. I remember that a year and a half after the study, when I came back from my gap year, I just tried to play and figure things out. I remember that I used to mark the bits I needed to think about with arrows, but not mark things I needed to do, because there was no time to practice.’

## CONCLUSION

In this exploratory study, the teacher used an indirect approach to teaching the use of PCs because she did not know whether they would be useful to a student at Maria’s level of training. Also, she wanted to avoid suggesting what kind of thoughts would be useful as PCs. As it turned out, Maria’s PCs did not differ in kind from those of her teacher (Chaffin et al., 2010; Lisboa et al., 2011, 2013), although they did reflect the different level of her musical understanding. It is possible that more explicit coaching might have helped Maria to memorize even more effectively. For example, Williamon and Valentine (2002) found that students who attended to musical structure earlier in their learning of a new piece, played better in their eventual performance. While the direction of causality in their study is unclear, our results suggest that encouraging Maria to attend to musical structure might have hastened the development of the mental organization needed for content addressable access to memory. We plan to examine this possibility in future studies by comparing the effectiveness of different ways of teaching memorization.

Caution in teaching students to use PCs as a memorization technique is suggested by the observation that experienced performers do not appear to regard PCs primarily as a means of memorizing. For the professional musicians who have been studied (see Chaffin, 2011), the role of PCs as memory retrieval cues appears to be largely incidental. Most PCs were developed in the service of other goals. The same is true of Maria. Most of Maria’s PCs concerned issues of expression (“feeling”), interpretation (“dynamics”), and basic technique (“repeated notes”). By reminding the musician of such goals in the midst of performance, PCs serve as retrieval cues. This result is, however, incidental to their main purpose. Sometimes, of course, memory itself may be the goal. If memory for a passage has been unreliable, a deliberately placed PC may solve the problem (see, Chaffin et al., 2010, Figure 5 and accompanying text for an example). Such deliberate use of PCs to solve memory problems appears, however, to be the exception rather than the rule.

Maria and her teacher were both surprised at the speed with which Maria memorized the piece, and the teacher was pleased that Maria played more musically than usual. For Maria, the discovery of PCs not only allowed her to perform from memory, it made her more confident about performing more generally. Previously, she had been reluctant to perform in public and avoided doing so whenever possible. After the completion of the study, Maria surprised her teacher by volunteering to perform, from memory, at the annual students’ concert. Her performance was a success and she went on to use the strategy of marking her thoughts on the score for other pieces, systematically making new reports as her practice progressed, saying, ‘this is a much more interesting type of practice than just repeating bits of the music.’ Thought reports and the PCs that they enabled, appear to have opened up new horizons in Maria’s music making.

## Conflict of Interest Statement

The authors declare that the research was conducted in the absence of any commercial or financial relationships that could be construed as a potential conflict of interest.
